# Melanin Distribution in Human Skin: Influence of Cytoskeletal, Polarity, and Centrosome-Related Machinery of *Stratum basale* Keratinocytes

**DOI:** 10.3390/ijms22063143

**Published:** 2021-03-19

**Authors:** Irene Castellano-Pellicena, Ciaran G. Morrison, Mike Bell, Clare O’Connor, Desmond J. Tobin

**Affiliations:** 1 The Charles Institute of Dermatology, School of Medicine, University College Dublin, D04 V1W8 Dublin, Ireland; irene.castellanopellicena@ucd.ie; 2 Centre for Chromosome Biology, School of Natural Sciences, National University of Ireland Galway, H91 W2TY Galway, Ireland; Ciaran.Morrison@nuigalway.ie; 3 Walgreens Boots Alliance, Nottingham NG90 1BS, UK; Mike.Bell@boots.co.uk (M.B.); Clare.OConnor@boots.co.uk (C.O.); 4 The Conway Institute of Biomolecular and Biomedical Research, University College Dublin, D04 V1W8 Dublin, Ireland

**Keywords:** melanin distribution, epidermis, *Stratum basale* keratinocytes, ex vivo human skin, skin phototype, centrosome, microtubules, centriolar satellites, actin

## Abstract

Melanin granules cluster within supra-nuclear caps in basal keratinocytes (KCs) of the human epidermis, where they protect KC genomic DNA against ultraviolet radiation (UVR) damage. While much is known about melanogenesis in melanocytes (MCs) and a moderate amount about melanin transfer from MC to KC, we know little about the fate of melanin once inside KCs. We recently reported that melanin fate in progenitor KCs is regulated by rare asymmetric organelle movement during mitosis. Here, we explore the role of actin, microtubules, and centrosome-associated machinery in distributing melanin within KCs. Short-term cultures of human skin explants were treated with cytochalasin-B and nocodazole to target actin filaments and microtubules, respectively. Treatment effects on melanin distribution were assessed by the Warthin–Starry stain, on centrosome-associated proteins by immunofluorescence microscopy, and on co-localisation with melanin granules by brightfield microscopy. Cytochalasin-B treatment disassembled supra-nuclear melanin caps, while nocodazole treatment moved melanin from the apical to basal KC domain. Centrosome and centriolar satellite-associated proteins showed a high degree of co-localisation with melanin. Thus, once melanin granules are transferred to KCs, their preferred apical distribution appears to be facilitated by coordinated movement of centrosomes and centriolar satellites. This mechanism may control melanin’s strategic position within UVR-exposed KCs.

## 1. Introduction

Melanin is predominantly restricted to the *basal* layer (or *Stratum basale*) of the human epidermis, where it is found in both melanocytes (MCs), the cells that make melanin, and in nearby keratinocytes (KCs), the cells of the epidermis that accept melanin. Melanin granules tend to be distributed in an ultraviolet radiation (UVR)-protective manner in KCs [1,2], forming supra-nuclear caps that co-localise with the cytoskeletal motor protein dynein [3]. We recently reported that melanin granule distribution in human skin is regulated primarily within in the *Stratum* (*S.*) *basale* of the epidermis, where they accumulate (and are largely, and remarkably, retained) via an asymmetric mode of organelle distribution [4]. This view challenged a long-held dogma, which explained a histologically apparent “depletion” of melanin in the epidermis above the *S. basale* as “degradation” of the melanin biopolymer, for which no convincing biochemical evidence has yet been advanced [5,6]. Instead, most melanin within dividing progenitor *S. basale* KCs appears to be inherited by the non-stratifying daughter KC. As a result, only a minor portion of melanin transits to the *S. spinosum* of the human epidermis via the stratifying daughter KC [4]. This mode of melanin distribution can explain the long-appreciated concentration of melanin within the *S. basale* and does so without invoking degradation of the super-resilient eumelanin indole biopolymer within an eminently degradable proteinaceous melanosomal capsule [7,8].

The transport of melanosomes inside epidermal MCs has been extensively studied and there are several excellent recent reviews on this topic [9,10]. The “highways and local roads” model suggests that melanosomes are transported along microtubules (MTs) between the MC centre and periphery (i.e., so-called long distances), before the melanosomes transition via short-range movements along the actin network [11]. This model has evolved in complexity into the “cooperative capture” model [10,12]. Recently, a “centrifugal and centripetal” model has been proposed, which envisions a role for actin also in long distance movements [13,14,15]. Nevertheless, all models agree on a role for both MTs and actin, together with their respective motor proteins, in melanosome movement within the epidermal MC.

Motor proteins are key molecules involved in the movement of organelles, including melanosomes, along the cytoskeleton. For example, Myosin Va can facilitate unidirectional movement of melanin along actin [12,16,17,18], while organelle transport via MTs undergoes both retrograde (to the –ve end of MT/cell nucleus) and anterograde (to the positive end of MTs/cell periphery) movement [19]. Retrograde melanosome transport along MTs is regulated by melanoregulin and Rab36, by interacting with the dynein–dynactin motor complex [20,21], while anterograde transport occurs through kinesins [22,23,24]. The motor protein Ninein-like [25], encoded by the *NINL* gene, has recently been implicated in melanosome transport and is variably expressed in *S. basale* epidermis of human skin according to skin colour (using L*), with its highest expression in medium pigmented skin followed by highly pigmented skin [25].

While progress on intra-MC melanosome transport has been impressive, our knowledge of melanin transport from MC to KC, and especially thereafter within the KC itself, is still relatively poor. The latter steps in the melanin journey are especially important for understanding how melanin distribution in the human epidermis is regulated, not only in normal skin melanin where it offers a protective strategy against skin cancer, but also in hypo- or hyper-pigmentary clinical conditions [2]. Some insights of intra-epithelial cell melanin granule choreography may be gleaned from the retinal pigmented epithelium (RPE) [26]. While both RPE and KCs are epithelial cells, the former is distinctive as RPE cells synthesise melanin themselves, albeit during a very time-limited period of embryogenesis [26]. Thereafter, melanin matures and accumulates in the apical compartment of the RPE cell. Despite this significant difference with KCs, Jiang et al. (2020) have shown how RPE melanosomes move along MTs until they reach the apical, actin-rich, RPE cell domain, a process dependent on motor proteins [27]. Thereafter, melanin remains for a lifetime, unless aging or disease perturb it. In the context of our study, RPE cells display significant cell polarity, with distinctive actin-rich apical cilia. Skin KCs may be less obviously polarised than RPE cells but still, they form a highly stratified epidermis with an organisation that requires the polarity of its constituent cells [28,29]. Another way skin KCs show cell polarity is via the characteristic distribution of melanin granules within “caps” and “parasols” in the apical KC domain (Figure 6).

MT networks are key contributors and responders to cell polarity [30], and the centrosome is considered the MT-organising centre (MTOC) in human somatic cells. The centrosome, consisting of two barrel-shaped centrioles embedded in a matrix of proteins known as the pericentriolar material, plays a key role in the transmission of cell polarity to daughter cells after cell division [31]. Pericentrin (PCNT) is an integral component of the pericentriolar material, to which it recruits multiple proteins (e.g., the MT nucleation component γ-tubulin), thereby ensuring proper centrosome and mitotic spindle formation. In this way, the centrosomes are responsible for the uninterrupted progression of symmetric and asymmetric cell divisions [32]. Another essential, but well-understood, part of the MT cytoskeletal machinery is the centriolar satellites. Centriolar satellites are non-membranous cytoplasmic granules, located around the centrosome (and the basal body of primary cilia), and are involved in the transport of proteins towards the centrosome and primary cilium [33]. PCM1 (Pericentriolar material 1) was the first centriolar satellite protein identified [34], followed more recently by others [35]. Importantly, centriolar satellites exhibit cell cycle-dependent assembly and disassembly [36].

In this study, we aimed to explore the role of the MT/actin cytoskeleton and associated centrosomal machinery in melanin distribution and localisation within KCs of the human epidermis, with a primary focus on the role of *S. basale* KC polarity in melanin distribution in human skin.

## 2. Results

We employed a small molecule approach to intervene in the dynamics of MT (via nocodazole) and actin (via cytochalasin B) [37] in KCs using our previously described ex vivo full-thickness human skin histoculture model [38]. While we have also examined epidermal KCs and MCs in vitro using mono-layer co-cultures [4,39] and pigmented epidermis 3D skin equivalents [4], we have concluded that these approaches may be limited in their capacity to faithfully recapitulate normal human epidermis cell dynamics. Specifically, we consider the principal deficiency of the aforementioned is the challenge of establishing and retaining epidermal cell polarity (data not shown), which we consider key to interrogating the fundamental biology of melanin dynamics in human skin. Thus, the approach adopted in the current study allows us to study melanin distribution and localisation not only in a 3D context, but also in one where it was possible to maintain most characteristics of human epidermis for a limited time ex vivo. Using this approach, we first examined whether MTs play a significant role in the distribution of melanin in KCs of the *S. basale*.

### 2.1. Microtubules Are Involved in the Localisation of Melanin into Supranuclear Caps in S. basale KCs

Nocodazole is widely used in biomedical research to depolymerise microtubules, including in melanin movement studies [15] and epidermal proliferation [40]. Here, we used this small molecule to study MT behaviour in *S. basale* KCs, exploring melanin localisation with the help of our recently re-introduced Warthin–Starry (WS) stain for melanin detection SPTII-III [41].

**Figure 1:**
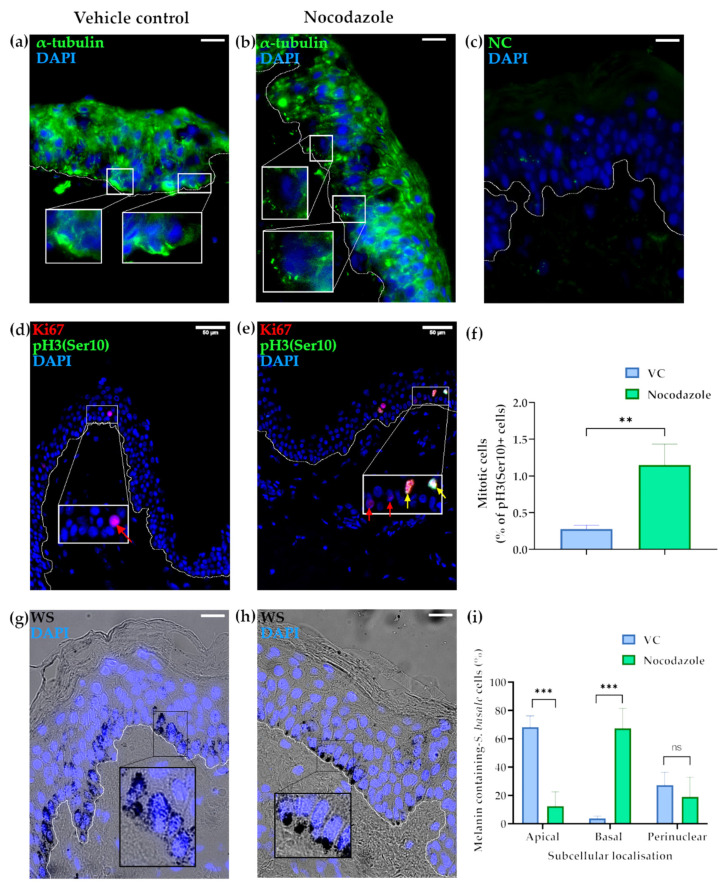
Microtubule depolymerisation (nocodazole treatment) influences the subcellular localisation of melanin aggregation in *Stratum basale* KC of human ex vivo skin epidermis. (**a**) Localisation of α-tubulin (green) showing microtubules in VC- and (**b**) nocodazole-treated ex vivo skin tissue after 24 h in culture; scale bar = 20 µm. (**c**) Negative control (omission of primary antibody) showed lack of non-specific binding. (**d**) Proliferating (Ki67 in red) and mitosis-stalled (pH3(Ser10) in green) KCs in VC- and (**e**) nocodazole-treated human ex vivo skin after 24 h in culture; scale bar = 50 µm as stated in the figure. (**f**) Percentage (%) of pH3(Ser10)-positive KCs per *S. basale* KC in VC- and nocodazole-treated cells. ** shows *p*-value < 0.01 in an unpaired *t*-test. (**g**) Warthin–Starry stain shows melanin localisation in vehicle control- (VC) and (**h**) nocodazole-treated ex vivo skin tissue after 24 h in culture; scale bar = 20 µm. Nuclei were counterstained with 4′,6-diamidino-2-phenylindole (DAPI in blue). Representative images from three individual donors. (**i**) Quantification of subcellular localisation of melanin. Graph shows the percentage (%) of *S. basale* cells with apical, perinuclear, and basal located melanin granules. Data are the mean ± SD of three independent biological experiments (*n* = 3 donors; 58–110 cells/replicate), *** indicates *p*-value < 0.001 and ns indicates not significant in a two-way ANOVA test.

Our readouts for the successful disruption of MTs by nocodazole was the latter’s impact on KC α-tubulin expression and on the progress to completion of KC mitosis in the ex vivo human skin post-treatment. Nocodazole-treated skin lacked long α-tubulin fibres, which were instead replaced by isolated, punctate α-tubulin-positive staining, especially in the *S. basale*, suggesting disrupted polymerisation of α-tubulin during the formation of the MTs (Figure 1a–c). The number of mitotic cells was assessed using the mitosis-specific marker pH3(Ser10) [42]. Nocodazole-treatment resulted in the increased detection of mitotic cells, indicating that disrupted MT polymerisation induced mitotic arrest, i.e., prevented those *S. basale* KCs that have started mitosis during the period of drug exposure from proceeding to completion of cell division, i.e., cytokinesis. Indeed, the fraction of mitotic KCs per total *S. basale* cells was significantly higher in nocodazole-treated skin in comparison to the vehicle-treated control (Figure 1d–f).

Melanin distribution in the vehicle control (VC)-treated skin showed granules predominantly present in the *S. basale* of the epidermis (Figure 1g), as occurs in vivo [2,4]. Moreover, the intracellular localisation of melanin was predominantly restricted to the apical domain of *S. basale* KCs. Specifically, melanin was aggregated into supra-nuclear “caps” (Figure 1g and Figure 6) in almost 70% of pigmented *S. basale* KCs. By contrast, melanin was almost absent from the basal domain of the KCs (<4% of cells), while the remaining approximately 25% of KCs had some melanin dispersed throughout the cell (Figure 1c). Nocodazole depolymerisation of MTs resulted in a dramatic alteration in the distribution of melanin granules inside *S. basale* KCs at the end of this short 24 h period of ex vivo culture. MT disruption caused a flipping of the melanin aggregate caps from the apical to the basal domains of the KC so that melanin was not adjacent to the basement membrane zone (BMZ) of the epidermis, i.e., close to the papillary dermis (Figure 1b). Approximately 10% of KCs still exhibited an apical domain preference for at least some melanin at the end of this short (24 h) histoculture period. The extent of non-aggregated or perinuclear melanin was not significantly different in nocodazole-treated and VC-treated tissues (Figure 1i). A broadly similar melanin distribution was found after 48 h of treatment (Supplementary Figure S1).

As melanin movement within MCs is in part mediated via actin, we next assessed the impact of disrupting actin polymerisation on melanin movement within epidermal KCs.

### 2.2. The Actin Cytoskeleton Is Involved in Melanin Granule Aggregation in S. basale KCs

**Figure 2:**
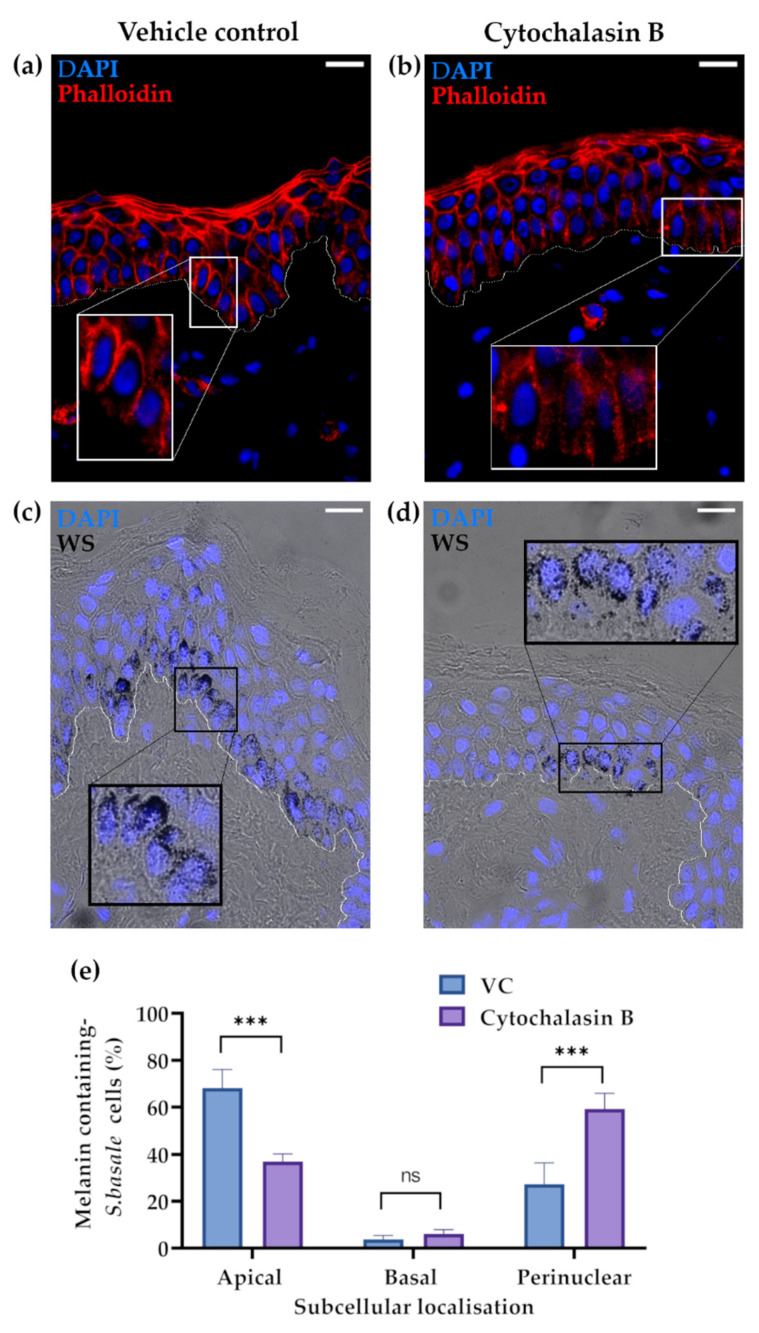
Reduced actin polymerisation after cytochalasin B treatment affects melanin aggregation in the *S. basale* KCs in human skin ex vivo. (**a**) Polymerised actin (phalloidin staining) is shown in red in VC- and (**b**) cytochalasin B-treated skin after 24 h in culture. Nuclear counterstain (DAPI) is shown in blue. (**c**) Warthin–Starry stain shows melanin localisation in VC- and (**d**) cytochalasin B-treated ex vivo skin after 24 h in culture. Scale bar = 20 µm. The basement membrane zone is shown by a white dotted line. (**e**) Quantification of subcellular localisation of melanin. Graph shows the percentage (%) of *S. basale* cells with apical, perinuclear, and basal located melanin. Data are the mean ± SD of three independent biological experiments (*n* = 3 donors; 61–131 cells/replicate). *** indicates *p*-value < 0.001 and ns indicates not significant in a two-way ANOVA test.

Cytochalasin B is a small drug widely used to impede actin polymerisation [43,44]. The impact of cytochalasin B treatment in our ex vivo human skin culture was assessed by phalloidin staining, which reflects cellular levels of polymerised actin. Polymerised actin filaments were shorter in *S. basale* KCs after cytochalasin B treatment (Figure 2b) compared to VC-treated skin (Figure 2a). This effect appeared to be restricted to the *S. basale*, as actin was similarly and strongly detected in KCs above, including in supra-basal KCs of both VC- and cytochalasin B-treated skin (Figure 2a,b). We next assessed the impact of actin polymerisation disruption on melanin movement within epidermal KCs. Cytochalasin B treatment significantly reduced apical domain distribution of melanin granules in human ex vivo skin tissue (SPTII-III) from 70% (VC-treated control) to approximately 30% (Figure 2e). When melanin was still present in the apical domain of cytochalasin B-treated KCs, its aggregation into supra-nuclear melanin caps was less commonly observed (Figure 2c,d). Similar melanin distribution was found after 48 h of treatment (Supplementary Figure S2).

With changes to MT and actin-associated cytoskeleton shown to influence melanin distribution in human epidermis (see above), we next examined whether the cell’s MT organising centre (MTOC), the centrosome, is also implicated in melanin distribution in the human epidermis.

### 2.3. The Centrosome, Centriolar Satellites and Melanin Granules Show a Coordinated Movement

To assess whether melanin granule movement within progenitor KCs is coordinated by the centrosome and its associated centriolar machinery, we examined the positional changes of key related proteins, including PCNT (centrosome) and PCM1 (centriolar satellites), after nocodazole disruption of the cytoskeleton in *S. basale* KCs.

**Figure 3:**
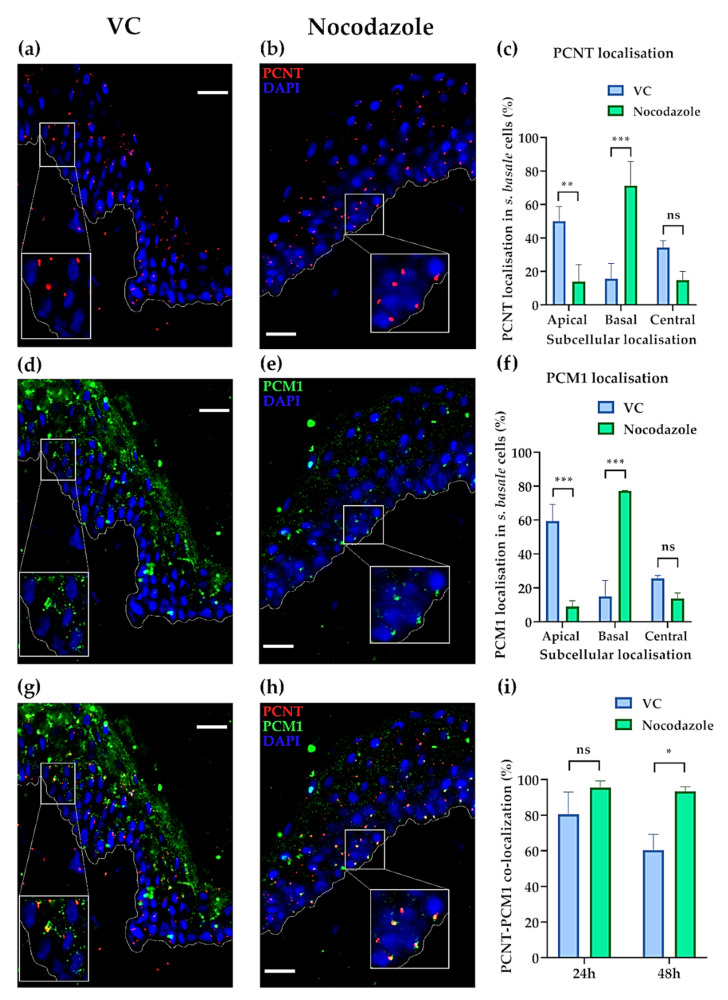
Microtubule depolymerisation (after nocodazole treatment) affects the subcellular localisation of the centrosome (PCNT) and centriolar satellites (PCM1) in *S. basale* KCs in human skin ex vivo. (**a**) Localisation of the centrosome (red) in VC- and (**b**) nocodazole-treated ex vivo skin after 24 h in culture. (**c**) Quantification of subcellular localisation of centrosome. Graph shows the percentage (%) of *S. basale* KCs with apical, basal and central PCNT localisation. ** indicates *p*-value < 0.01, *** indicates *p*-value < 0.001 and ns indicates not significant in a two-way ANOVA test (*n* = 3 donors; 33–55 PCNT dots/replicate). (**d**) Localisation of centriolar satellites (green) in VC- and (**e**) nocodazole-treated ex vivo skin after 24 h in culture. (**f**) Quantification of centriolar satellite localisation. Graph shows the percentage (%) of PCM1 satellite clouds with apical, basal and central localisation. *** indicates *p*-value < 0.001 and ns indicates not significant in a two-way ANOVA test (*n* = 3 donors, 25–45 PCM1 clouds/replicate). (**g**) Co-localisation (orange/yellow) of centrosome (PCNT, red) and centriolar satellites (PCM1, green) in VC- and (**h**) nocodazole-treated ex vivo tissues after 24 h in culture. (**i**) Quantification of centrosome co-localisation with centriolar satellites. Graph shows the percentage (%) of co-localisation in *S. basale* KCs. * indicates *p*-value < 0.05, ns indicates not significant using one-way ANOVA (*n* = 3 donors, 22–60 cells/replicate).

Nocodazole treatment caused a striking movement of the centrosome (PCNT) from the apical to the basal domains of *S. basale* KCs, with a basal centrosome position seen in the majority (~70%) of cases. By contrast, the majority of centrosomes in VC-treated skin showed a retention of their original apical domain position (Figure 3a–c). Moreover, nocodazole treatment caused a similar translocation of the centriolar satellites (PCM1), from the apical to basal domain of the KCs. While the satellites exhibited a characteristic [33,45] punctate “cloud” distribution around the centrosome in the apical domain of KCs in VC-treated skin (Figure 3d), nocodazole treatment caused these centriolar satellites to adopt a much more restricted localisation around the centrosome when moved to the basal domain of the KC (Figure 3e). In this way, centriolar satellite (PCM1) movement paralleled that of the centrosome (PCNT) after nocodazole treatment (Figure 3f). A similar effect of nocodazole treatment on the positions of the centrosome and centriolar satellites was observed when human skin was treated for 48 h in ex vivo histoculture (Supplementary Figure S3). The high level of coordination between these two structures was evidenced by co-localisation of PCNT and PCM1 in the vast majority of KC in both VC- and nocodazole-treated tissues after 24 h in culture. However, after 48 h in culture, we observed that the co-localisation was even higher in nocodazole-treated skin (Figure 3g–i).

To assess whether this coordinated movement of centrosomes and their associated centriolar satellites may also influence the observed treatment-affected movement of melanin granules within *S. basale* KCs in these histocultures, we attempted to assess whether the modulated positioning of the latter melanin granules matched that of the centrosome/centriolar satellites. This was technically challenging, especially in low pigmented SPTII and III skin, because of the difficulty of combining Warthin–Starry with immunofluorescence staining. Still, we were able to determine that melanin granule and PCNT (centrosome) expression indeed shared a common intracellular localisation in both VC- and nocodazole-treated tissues (Supplementary Figure S4), and therefore, indirectly allowed us to correlate overall melanin and centrosome co-localisation in these *S. basale* KCs.

We also assessed whether the observed change in melanin behaviour, seen after this small molecule treatment, was due to (drug) treatment effects on KC differentiation within the histocultures. The similar expression of the early differentiation marker keratin 10 (KRT10) in the *S. basale* KCs of both nocodazole-, cytochalasin B, and VC-treated skin (Supplementary Figure S5) suggests that we were observing direct effects of both MT and actin cytoskeleton on melanin movement within the *S. basale* KC. Thus, the absence of KC differentiation effects during the very short time course of our experiments supports the usefulness of this model, with or without drug treatments.

Melanin biology in low and high pigmented human skin types shows several distinctive features, not least in the size and processing of their melanosomes/melanin granules [2]. Therefore, we were keen to examine how the centrosome/microtubule machinery is organised in skin of different intrinsic melanin levels, and secondly, whether this may affect how skin phototype may impact melanin granule distribution in the epidermis.

### 2.4. The Status of the Centrosome/Microtubule Machinery in Low versus High SPT Human Skin In Situ

**Figure 4:**
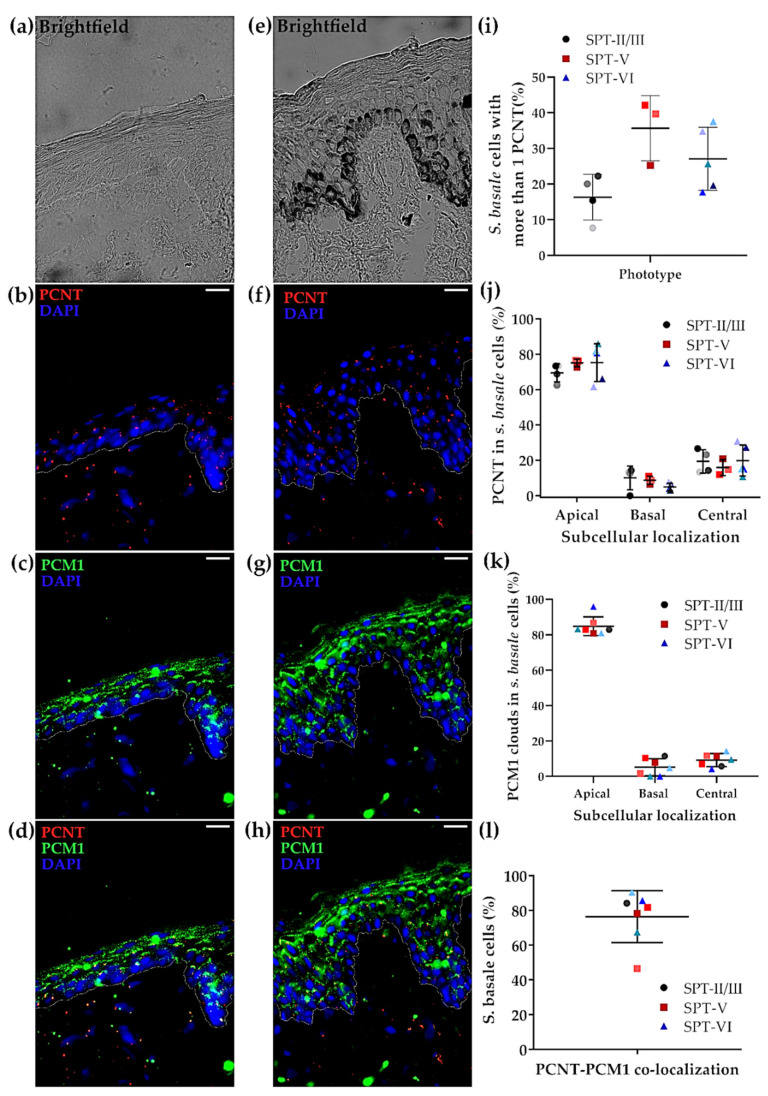
Centrosome (PCNT) and Centriolar satellites (PCM1) in low vs. high SPT skin. Representative images (scale bar = 20 µm) of SPT-II/III human skin tissue; (**a**) brightfield image showing low level of pigmentation, (**b**) in situ localisation of centrosomes (PCNT in red) and (**c**) centriolar satellites (PCM1 in green). (**d**) Merged image of b and c. Representative images of SPT-VI human skin tissue; (**e**) brightfield image showing high level of pigmentation, (**f**) in situ localisation of PCNT in red and (**g**) PCM1 in green. (**h**) Merged image of f and g. (**i**) Quantification of *S. basale* KCs with more than one centrosome. Graph shows the percentage (%) of *S. basale* KC with more than one centrosome (i.e., PCNT dot) in SPT-II/III (black), V (red) and VI (blue) human skin. No significant differences were found using one-way ANOVA test (each dot represents the summary data for one donor, *n* = 12). (**j**) Quantification of subcellular localisation of centrosomes. Graph shows the percentage (%) of the total centrosomes (PCNT dots) that showed an apical, basal and perinuclear/central localisation in SPTII/III (black), SPTV (red) and SPTVI (blue) human skin. No significant differences were found using multiple *t*-test (each dot represents the summary data for one donor, *n* = 12). (**k**) Quantification of subcellular localisation of centriolar satellites (PCM1). Graph shows the percentage (%) of the total PCM1 clouds with an apical, basal and perinuclear/central localisation in SPTII/III (black), SPTV (red) and SPTVI (blue) human skin (each dot represents the summary data for one donor, *n* = 7). (**l**) Number of *S. basale* KC (%) showing PCNT co-localisation with PCM1 (each dot represents the summary data for one donor, *n* = 7). Each individual donor is represented using the same colour shade in all graphs of this figure.

The localisation of centrosome (PCNT) and centriolar satellites (PCM1) was compared in lightly (i.e., SPT I/II) and darkly pigmented skin in situ (i.e., SPT V/VI) using brightfield microscopy (Figure 1a,e), given that melanin granules are much more numerous outside the *S. basale* (i.e., in the supra-basal layers of the epidermis) in darkly versus lightly pigmented human epidermis [4]. The most common localisation for the centrosome and centriolar satellites was in the apical domain of the *S. basale* KCs in vivo (Figure 4b,c,f,g and quantified in Figure 4j,k), i.e., similar to what we observed in the VC-treated ex vivo tissue (Figure 3). Indeed, no significant differences were found in either the number (Figure 4i) or localisation (Figure 4j) of PCNT dots between lightly (SPTII/II) and darkly (SPTV/VI) pigmented skin. Moreover, around 80% of *S. basale* KCs showed co-localisation between PCNT and PCM1 stainings of both light and darker pigmented skin tissues in vivo (Figure 4l).

### 2.5. Melanin Co-Localises with the Centrosome (PCNT) in Both Light and Dark Skin Phototypes In Vivo

**Figure 5:**
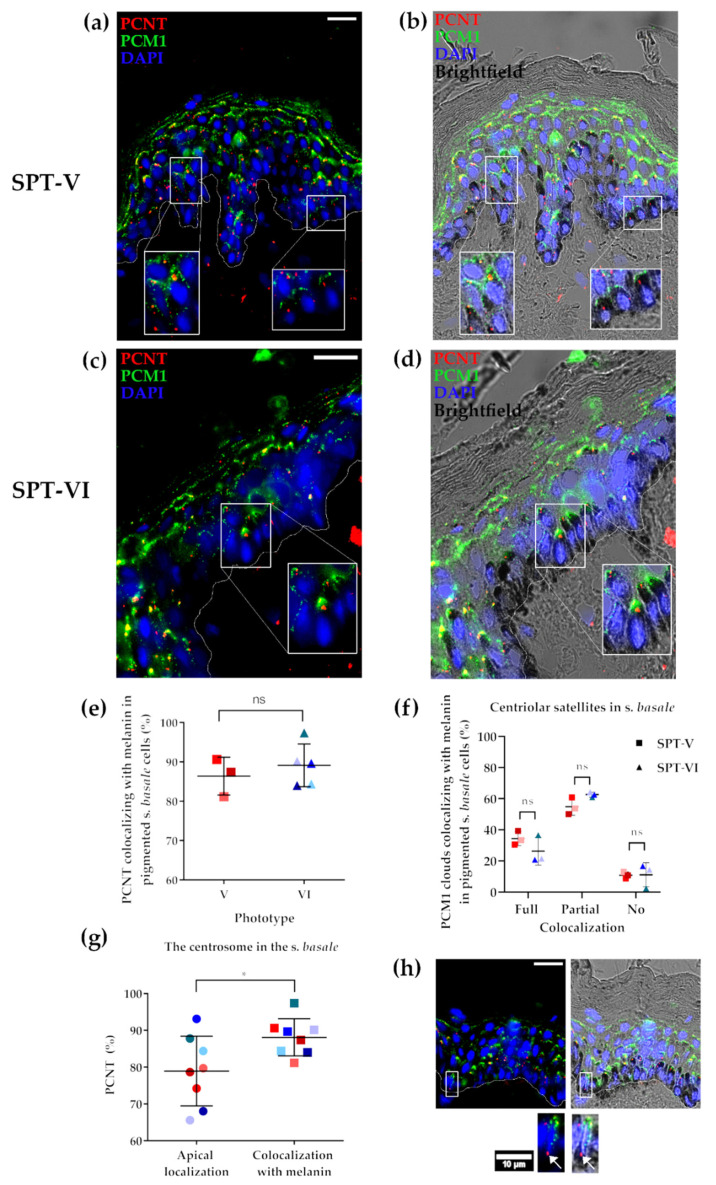
Co-localisation of centrosomes (PCNT) and centriolar satellites (PCM1) with melanin granules in highly pigmented human skin (SPT V and VI). (**a**) Representative image of PCNT (red) and PCM1 (green) localisation in SPT V skin. (**b**) Image a merged with brightfield microscopy view. (**c**) Representative image of PCNT (red) and PCM1 (green) localisation in SPT VI skin. (**d**) Image (**c**) merged with brightfield microscopy view. (**e**) Percentage (%) of pigmented *S. basale* KC showing co-localisation of PCNT and melanin; ns indicates not significant in an unpaired non-parametric *t*-test (Mann–Whitney). (**f**) Percentage (%) of PCM1 clouds (centriolar satellites) showing full, partial or no co-localisation with melanin; ns indicates not significant in a two-way ANOVA. (**g**) Percentage (%) of centrosomes (PCNT) showing an apical localisation in pigmented *S. basale* cells in comparison with the percentage (%) of centrosomes co-localising with melanin independently of its subcellular localisation; * indicates *p* value < 0.05 in a paired non-parametric *t*-test (Wilcoxon, *n* = 8 donors). For e, f and g, each coloured dot represents a different donor, red shades indicate SPTV (*n* = 3) and the blue shades indicate SPT-VI (*n* = 3–5). (**h**) Example of *S. basale* KC showing basal PCNT co-location with melanin in SPT-VI skin. Scale bar = 20 µm unless indicated otherwise. Nuclei were counterstained with DAPI. Basement membrane is shown with a dotted white line.

The association of melanin granules with the centrosome and centriolar satellites was analysed in the *S. basale* KCs of highly pigmented (SPT-V and SPT-VI) human skin, where melanin localisation, after Warthin–Stary staining, can be directly analysed by brightfield microscopy (Figure 4a,e). Melanin co-localisation with the centrosome was found to occur in nearly all (~90%) *S. basale* KCs in vivo (Figure 5b,d,e). To assess if the occurrence of this co-localisation was confined to the apical domain of the KC, we compared total detectable centrosomes in the apical domain of KCs with total detectable centrosomes that co-localised with melanin anywhere in the KC. The proportion of total centrosomes that co-localised with melanin was always statistically significantly higher than the number of centrosomes restricted to the apical domain of the KCs (Figure 5g,h), indicating a coordinated (i.e., non-random) relationship between the centrosome machinery and melanin in the human epidermis. As mentioned above, centriolar satellites tend to be distributed in human skin KCs in the form of diffuse “clouds” (Figure 4). Interestingly, full or complete co-localisation of PCM1 satellite “clouds” with melanin granules occurred in ~30% of KC, while partial co-localisation with melanin was observed in ~60% of KC in SPT-V/VI skin (Figure 5b,d,f).

While melanin granule distribution in *S. basale* KCs of the human epidermis exhibited an apparently coordinated, domain-specific, localisation with both the centrosome and centriolar satellites, this was not seen for the expression of key motor proteins dynactin (DNCT1) and ninein-like (NINL), which were observed throughout the KC cytoplasm (Supplementary Figure S6). This finding indicates no specific co-localisation of these proteins with melanin granules in the steady-state epidermis.

## 3. Discussion

For perhaps more than a century, discussion on the fate of melanin granules in the human epidermis, after their transfer from MC to KC, has focused on some form of melanin “degradation”. More recently, this has included reference to autophagy-mediated melanin degradation [46,47,48,49], which is purported to be a consequence of KC differentiation and stratification [50]. In the absence of empirical supporting evidence, this view has proved insufficient to explain the biology of melanin distribution in the human epidermis [4,5,6], particularly given that no mammalian enzyme chemistry has yet been described that can obliterate the highly stable indole biopolymer that is eumelanin [7]. Our laboratory has, therefore, sought to reassess the basis for the striking basal layer predominance of melanin in the human epidermis, and recently proposed a wholly different explanation for the fate of melanin within human skin [4]. Our findings to date suggest that the pattern of histochemically detectable melanin in human epidermis results from a preferential segregation of melanin cargo from the mitotic progenitor KC into the daughter cell that replaces the mother cell in the *S. basale*. As a result, only a minor fraction of melanin is “inherited” by the daughter KC that leaves the *S. basale* to differentiate and stratify. In this way, most (~70% in Caucasian epidermis) of the skin’s melanin is retained in the *S. basale* of the epidermis, where its UVR protection needs are greatest. This example of asymmetric organelle distribution, preferentially into one daughter cell of a progenitor KC in the *S. basale*, may in fact not be as rare of first though. It has been reported recently for mitochondria, albeit in vitro, where this strategy may be important for the maintenance of cell stemness [51]. Perhaps even more relevant to the melanosome (a lysosome-related organelle), this mode of organelle inheritance been also recently been reported for lysosomes during HaCaT keratinocyte cell division [52].

We know much about how melanin moves within MCs [9,10,12,13,15,18,20,23,24] and also about the multiple ways melanin leaves the MC to enter the KC [39,53,54,55]. We have started to explore how melanin remains in large part tethered to the *S. basale* [4], as can be seen so strikingly with routine melanin-staining protocols [41]. The current study focuses on how melanin granules distribute in the epidermis as crucially important UVR-protective, supra-nuclear caps [2]. We report here several new insights into the regulation of melanin homeostasis in the human epidermis by exploiting two small cytoskeleton-disrupting drugs, nocodazole and cytochalasin B, using our short-term ex vivo human full thickness skin histoculture model [38]. Nocodazole impacted on melanin distribution by disrupting microtubule (MT) polymerisation to alter the polarity of melanin localisation within KC, i.e., essentially flipping melanin aggregates from the apical to the opposite (basal) pole of *S. basale* KCs. The second drug, cytochalasin B, impacted on melanin granule aggregation by slowing down actin polymerisation. Importantly, we view the first scenario (i.e., the outcome of MT depolymerisation) as being reminiscent of the re-location of melanin observed during asymmetric *S. basale* KC mitosis in situ, where one daughter replaces the mother cell in the *S. basale*, while the remaining daughter stratifies and terminally differentiates [4]. Thus, we speculate that rearrangement of MTs during cell division and mitotic spindle formation facilitates the movement of melanin granules from the apical domain to the basal pole of the dividing KC, as appears to be modelled here in the nocodazole-treated epidermis.

Moreover, our data support the view that coordinated MT and actin cytoskeleton mechanisms work together to maintain the preponderance of eumelanin granules in the apical domain of the vulnerable proliferative KCs, where they are best placed to protect the latter from the damaging effects of UV radiation [2]. Perhaps unsurprisingly, similar cellular machinery (i.e., MTs and actin) for melanin movement also pertains to the MCs [9,56], although its function/purpose in the context of the melanosome (when in MCs) versus the melanin granule (when transferred into KCs) may vary. Our data did not show a change in localisation of the motor protein dynactin 1 (DNCT1) in the epidermis after treatment with nocodazole. However, we suggest that melanin granules will have already moved at the time of DNCT1 detection (i.e., end of incubation period) and after the presumptive period of motor protein action. Currently, the identity of motor proteins involved in melanin granule movement within the *S. basale* KCs remains unknown, although we hypothesise that different motor proteins may be involved in melanin movement within the KCs (versus within MCs), not least because of the autophagolysosomal compartment context of melanin granules in the KCs.

To reduce the possibility of ex vivo skin culture artefacts in our study, we analysed melanin localisation within the first 24 h of culture, when progenitor KCs in the *S. basale* were shown to remain proliferative and when even early KC differentiation (as assessed by K10) was not altered. Moreover, this first 24 h appeared to be sufficient to affect melanin granule movement within the *S. basale*.

A limitation of this study is its reliance on the use of an ex vivo human skin model, which does not allow for a wide range of biological read-outs to be performed. Other techniques may be useful to identify proteins directly interacting with melanin in KCs. For example, proteomics may identify specific proteins that interact with melanin granules within KCs, while live-cell imaging may help visualise melanin granule movement in concert with potential candidate molecular motors, etc. Unfortunately, such imaging techniques are extremely challenging to perform within 3D models. Therefore, we heavily relied on immunohistochemistry at specific timepoints for this study, which gave us a snapshot in time of melanin localisation rather than revealing the whole melanin movement process. As previously mentioned, we consider that cell polarity plays a key role in melanin movement in the human epidermis and it has proven very difficult to retain optimal cell polarity using 2D or even 3D skin reconstruction models [4]. Thus, an ex vivo human full-thickness skin histoculture model was chosen for this study, and despite its limitations in terms of variety of biological read-outs, we found that this model was very successful in mimicking in vivo epidermal cell polarity. Another limitation of our study relies on the use of cytochalasin B and nocodazole. Both drugs have a wide range of effects on the cell cytoskeleton. However, this study reveals, for the first time, melanin localisation and movement processes in epidermal keratinocytes, which will allow for future studies to have a more targeted approach.

The importance of the MT cytoskeleton in KC melanin movement was previously suggested by data showing the apical co-localisation of melanin with the motor protein, dynein, in human skin [3]. Here, we show that not only is the location of melanin granules affected by MT function, but so too was the behaviour of centrosomes and centriolar satellites, with melanin granules apparently moving in tandem with these structures from the apical to basal domain of epidermal KCs (Figure 2 and Figure 3). The centrosome exhibits an apical domain localisation in steady-state mouse epidermis [57], similar to our vehicle control-treated human tissues ex vivo. We show, for the first time, the apical subcellular localisation of centriolar satellites in human skin ex vivo (Figure 3) and in vivo (Figure 4). Centriolar satellites tend to spread throughout the cytoplasm by following the centrosome [58], and have been assessed in different cell types in culture (e.g., endothelial cells [59] or epithelial (retina) cells [45,60]). The intracellular localisation of centriolar satellites can be disrupted by either increasing their dispersion throughout the cytoplasm, or by causing them to accumulate around the centrosome [61]. In this current study, we observed that accumulation of centriolar satellites occurred when the skin was treated with the MT-depolymerisation drug, nocodazole (Figure 3). The change in centriolar satellite distribution towards accumulation instead of dispersion will probably have functional consequences in human skin, which remain to be elucidated.

The level of skin pigmentation (i.e., degree of melanisation) did not appear to significantly affect centrosome and centriolar satellite (co-)localisation (Figure 4) in the *S. basale* KC. Apical localisation of the centrosome and centriolar satellites does not, therefore, appear to be a consequence of degree of pigmentation, but rather of KC/epidermis polarity [62]. Our data on darkly pigmented skin epidermis in situ (SPTV and SPTVI) showed a very high degree of co-localisation of melanin granule aggregates with the centrosome in *S. basale* KCs, i.e., beyond simply sharing the general apical domain of the cell (Figure 5). Moreover, centriolar satellites also showed either full or partial co-localisation with melanin granules. Importantly, disruption of MTs induced a similar translocation in melanin granules, centrosomes and centriolar satellites towards the basal domain of the KCs in both pale and dark skin. Thus, we suggest that melanin granule re-localisation may be facilitated by the coordinated movement of the centrosome together with its associated centriolar satellites. Crucially, this may be an important mechanism to control melanin’s strategic positioning above the KC cell nucleus to regulate UVR protection. We are still at a very early stage in our understanding on how skin cell polarity influences skin pigmentation and should not over-extrapolate from either these data or data emerging from the (nocturnal) mouse [28,63,64]. Rising rates of human skin cancer globally attest to the pressing need to better understand how the essential human survival trait of skin pigmentation is regulated.

## 4. Materials and Methods

### 4.1. Human Skin Samples

Ethical approval was obtained (#LS-19–71, University College Dublin) (11th of September 2019) to collect anonymised human skin (SPT-II/III) after abdominoplasty or breast reduction surgery (Blackrock Clinic, Dublin, Ireland). Further highly pigmented skin samples were kindly provided by Prof. Rachel Watson (University of Manchester) [65] in accordance with local UK’s Human Tissue Authority Act (2006) regulations and Declaration of Helsinki principles. Samples were stored at −80 °C until use and are indicated below (Table 1).

### 4.2. Histoculture of Full-Thickness Human Skin Ex Vivo

Skin was transported from the clinic to the laboratory in cooled (4 °C) transport medium of DMEM (Sigma-Aldrich, Poole, Dorset, UK) with 10% FBS (Fisher Scientific, Loughborough, UK); and antibiotics (InvivoGen, Toulouse, France) and processed within 15 h of surgery into fat-free, rectangular skin tissue pieces (~0.8 mm^2^). The skin pieces were cultured in 12-Well Hanging Inserts (Millicell, MCEP12H48, Merck, Dorset, UK), with the following treatments for 24 and 48 h: 2 µM nocodazole (for inhibition of microtubule polymerisation) (Fisher Scientific, Goteborg, Sweden) and 10 µM cytochalasin B (for decreased rate of actin polymerisation) (Fisher Scientific, Goteborg, Sweden) in DMEM. Vehicle control (VC)-(including 0.1% DMSO (Sigma-Aldrich, Poole, Dorset, UK) used for the nocodazole and cytochalasin B incubations) treated skin samples were included as negative controls. Treatments were applied to the surface of the epidermis (1.5 mL/well), and tissues were cultured in the air–liquid interphase. The tissues were washed with PBS and flash-frozen in OCT medium (VWR; Dublin, Ireland) after 24 and 48 h (in duplicates).

### 4.3. Immunohistochemistry/Immunofluorescence

Six micrometre cryosections were cut using a cryostat (−27 °C), air-dried and fixed with acetone for 10 min at −20 °C. The effect of drug treatments on melanin localisation was examined with Warthin–Starry stain, as previously reported by our laboratory [41]. Vectashield Hardset antifade mounting medium with DAPI (Vector laboratories, H-1500, Vector Laboratories, Peterborough, UK) was used to mount the sections and to counterstain cell nuclei.

The following primary antibodies were used: mouse monoclonal antibodies against alpha-tubulin (clone B512; Merck, Darmstadt, Germany; T5168; 1:200); phospho-histone H3 Ser10 (ab14955, Abcam, Cambridge, UK 1:250); cytokeratin 10 (clone RKSE60; Invitrogen MAI-06319, 1:200); goat polyclonal antibodies against DCTN1/p150 glued (ab11806, Abcam Cambridge, UK 1:200); PCM1 (817; Dammermann and Merdes (2002) [36], 1:500); pericentrin (ab4448, Abcam, Cambridge, UK 1:200). Alexa 488-labelled goat (anti mouse, A32723; Invitrogen, Dublin, Ireland), Alexa 488-labelled donkey (anti goat, A32814; Invitrogen, Dublin, Ireland) and Alexa 555-labelled donkey (anti rabbit, A32794, Invitrogen, Dublin, Ireland) secondary antibodies were from Invitrogen and used at 1:200. Phalloidin iFluor 555 (Abcam, Cambridge, UK ab176756, 1:1000) was used to investigate actin.

**Figure 6:**
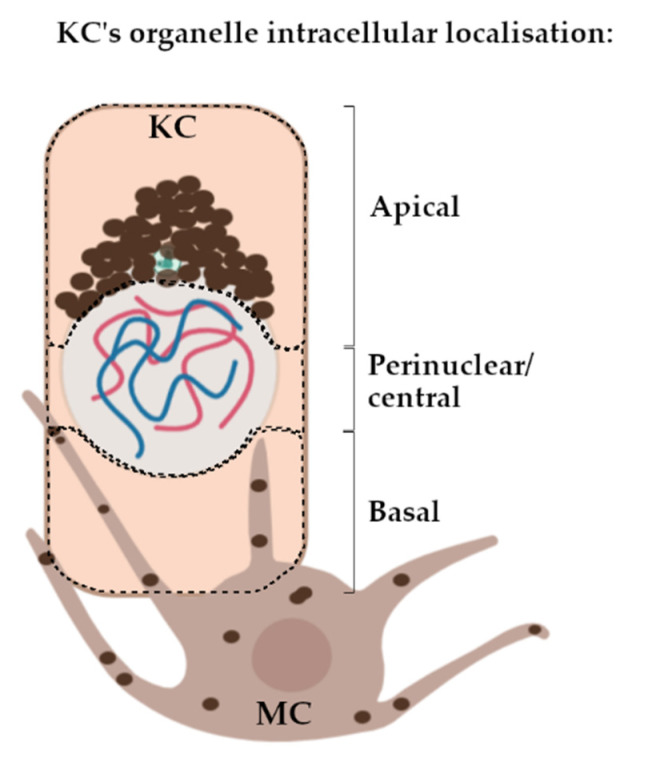
Methodology for assigning apical, perinuclear/central or basal subcellular localisation of KC’s organelles (i.e., melanin granules, PCNT and PCM1) in the *S. basale*. KC, keratinocyte; MC, melanocyte.

Immunostaining visualisation was performed using an IX83 Fluorescent Inverted Microscope (Olympus, Hamburg, Germany). Images were taken using the Olympus cellSens Software. Z-stack images were taken for centrosome (PCNT) and centriolar satellite (PCM1) localisation analysis (0.2 µm interval). Apical, basal and perinuclear localisations of melanin, PCNT and PCM1 in *S. basale* cells were determined according to Figure 6. Data were entered and processed in Excel. Statistical analyses, including *t*-test and one-/two-way ANOVA, were performed using GraphPad Prism (version 8.0.0 for Windows, GraphPad Software, San Diego, CA, USA). Specifics regarding the type of statistical analysis performed is indicated in the figure legends.

## Figures and Tables

**Table 1:** Information of human skin samples used for histoculture experiments or for low vs. high pigmentation comparisons in situ. (SPT—skin phototype; F—female; M—male).

SPT	Sex	Age (Years)	Histoculture Samples	In Situ
VI	F	25		x
VI	F	18		x
VI	F	77		x
VI	F	19		x
VI	M	25		x
V	F	21		x
V	F	27		x
V	F	22		x
II/III	F	46	x	
II/III	F	46	x	x
II/III	F	40		x
II/III	F	43	x	x
II/III	F	43		x

## Data Availability

Not applicable.

## Supplementary Materials

Supplementary Materials can be found at https://www.mdpi.com/1422-0067/22/6/3143/s1. Figure S1: Microtubules are involved in supranuclear cap melanin localisation in *s. basale* KCs—48h skin histoculture data. Figure S2: The actin cytoskeleton is involved in melanin granules clustering in *s. basale* keratinocytes—48h skin histoculture data. Figure S3: Basal centrosome (PCNT) and centriolar satellite (PCM1) localisation was confirmed after treatment of ex vivo human skin with nocodazole. 48h data. Figure S4: Co-localisation of melanin granules and centrosomes (PCNT) in both VC- and nocodazole-treated skin i.e., no statistically-significant differences in subcellular localisation detected. Figure S5: No difference in the subcellular localization of the microtubule-linked motor protein DNCT1 between VC-, cytochalasin B- or nocodazole-treated skin tissues. No difference in the expression of the epidermis differentiation maker, KRT10, between VC-, cytochalasin B- or nocodazol- treated skin tissues. Figure S6: Expression of the motor proteins DNCT1 (green) and NINL (red) was broadly similar in both high (SPT V) vs. low (SPT II) pigmented human skin.

## Abbreviations

DAPI	4′,6-diamidino-2-phenylindole
DNCT1	Dynactin 1
F	Female
KC	Keratinocyte
KRT10	Keratin 10
M	Male
MC	Melanocyte
MT	Microtubule
MTOC	MT-organising centre
NINL	Ninein-like
PCM1	Pericentriolar Material 1
PCNT	Pericentrin
RPE	Retinal pigmented epithelium
*S.*	*stratum*
SPT	Skin phototype
UVR	Ultraviolet radiation
VC	Vehicle control
WS	Warthin–Starry
