# Supplementary material for: Melanin Distribution in Human Skin: Influence of Cytoskeletal, Polarity, and Centrosome-Related Machinery of Stratum basale Keratinocytes
Source: Int J Mol Sci. 2021 Mar 19;22(6):3143. doi: 10.3390/ijms22063143 (PMC8003549; doi:10.3390/ijms22063143)
Supplement: Supplementary file 1 [file ijms-22-03143-s001.pdf]

Supplementary material

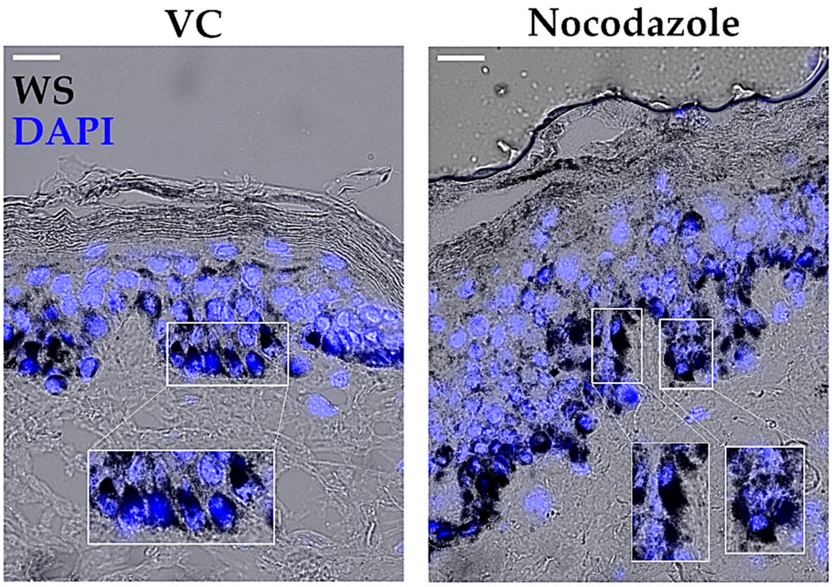

Supplementary Figure S1: Microtubules are involved in supranuclear cap melanin localisation in *s. basale* KCs – 48h skin histoculture data. SPT III donor (scale bar = 20 µm).

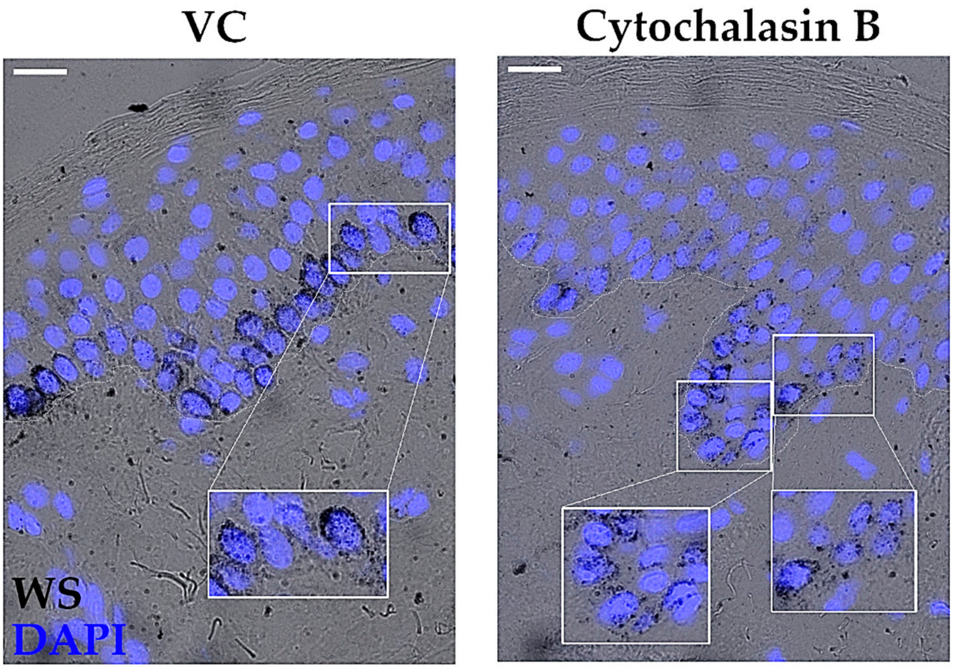

Supplementary Figure S2: The actin cytoskeleton is involved in melanin granules clustering in s. basale keratinocytes – 48h skin histoculture data. SPT II donor (scale bar = 20).

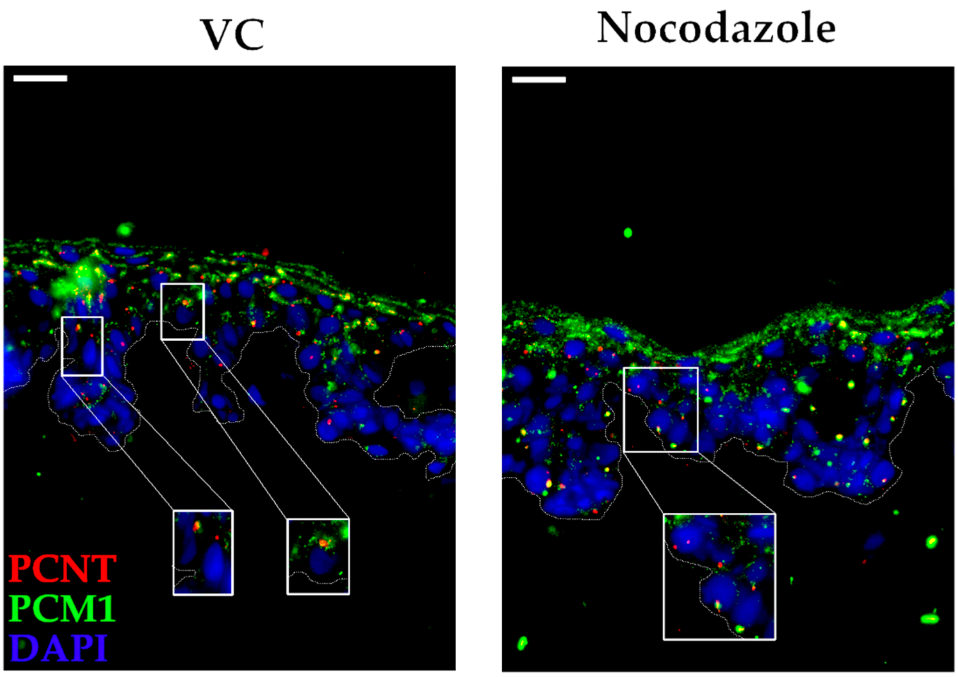

Supplementary Figure S3: Basal centrosome (PCNT) and centriolar satellite (PCM1) localisation was confirmed after treatment of ex vivo human skin with nocodazole. 48h data (scale bar = 20 μm).

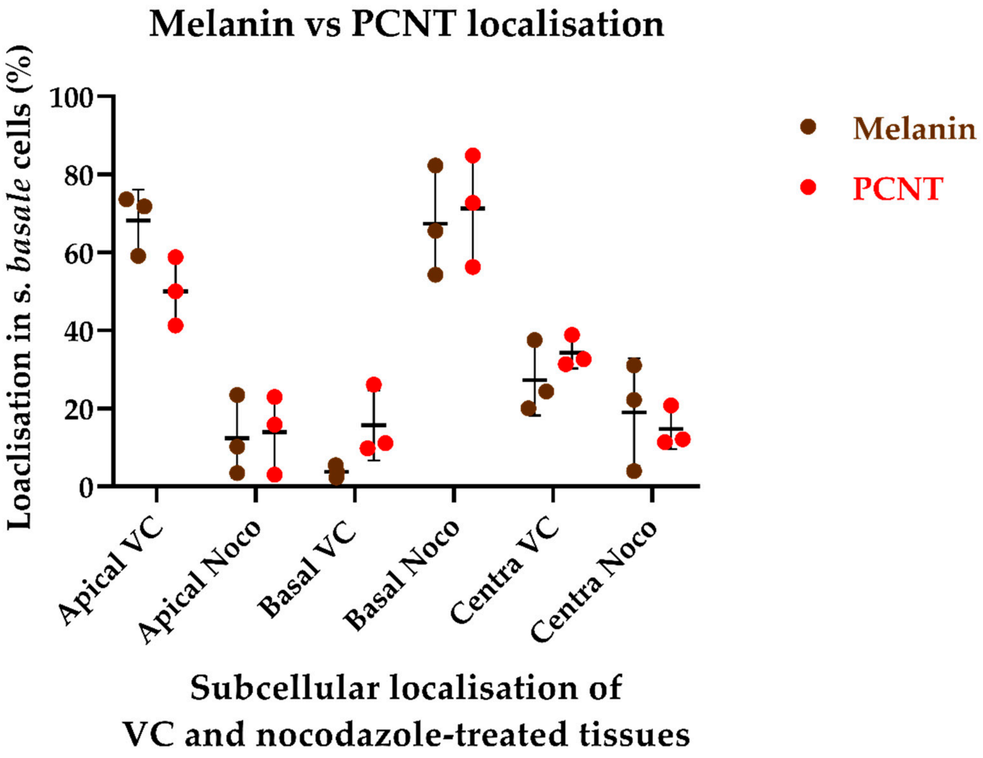

Supplementary Figure S4: Co-localisation of melanin granules and centorsomes (PCNT) in both VC- and nocodazole-treated skin i.e., no statistically-significant differences in subcellular localisation detected.

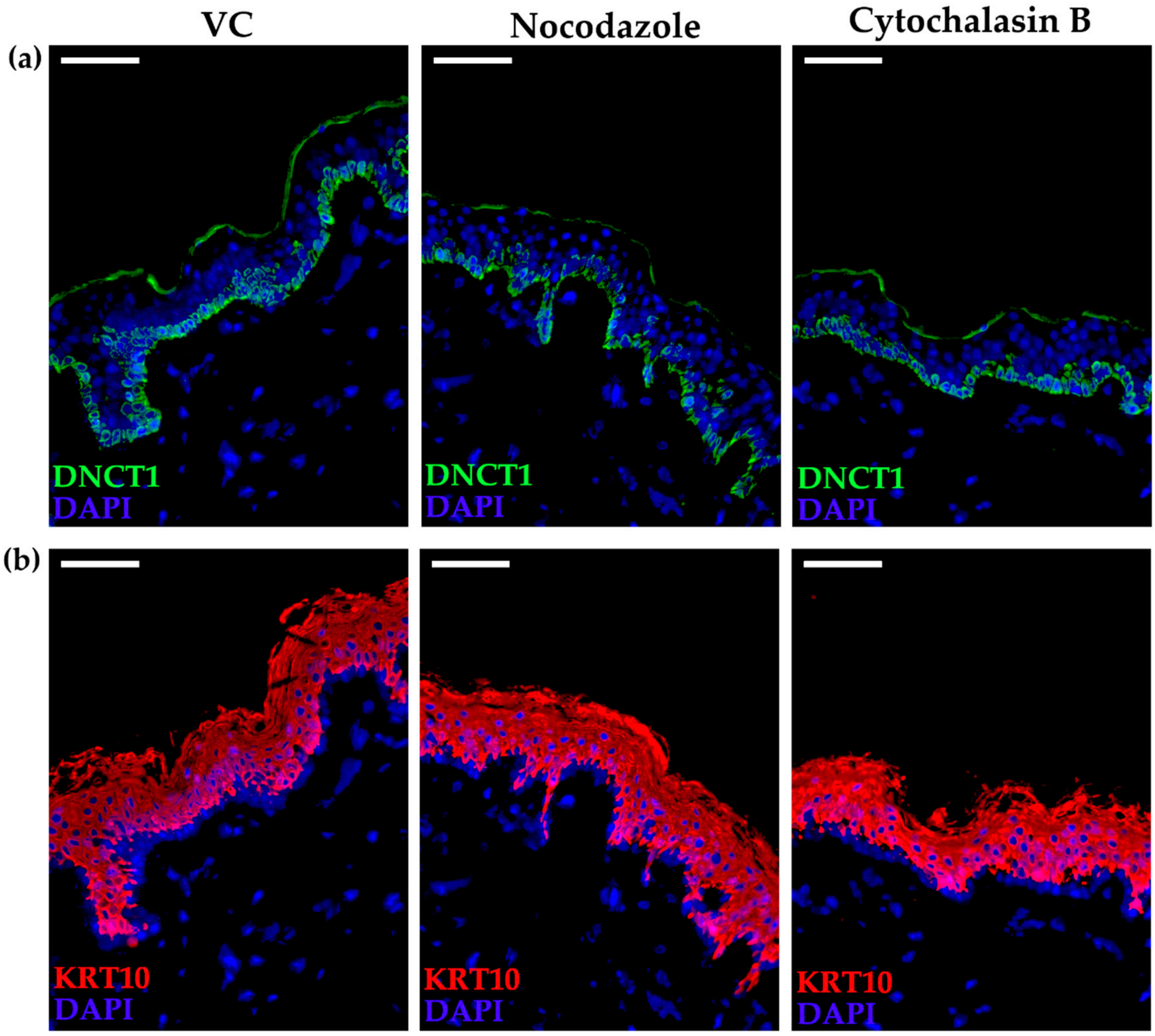

Supplementary Figure S5: No difference in the subcellular localisaiton of the microtubule-linked motor protein DNCT1 between VC-, cytochalasin B- or nocodazole-treated skin tissues. No difference in the expression of the epidermis differentiation maker, KRT10, between VC-, cytochalasin B- or nocodazol- treated skin tissues (scale bar = 50 μm).

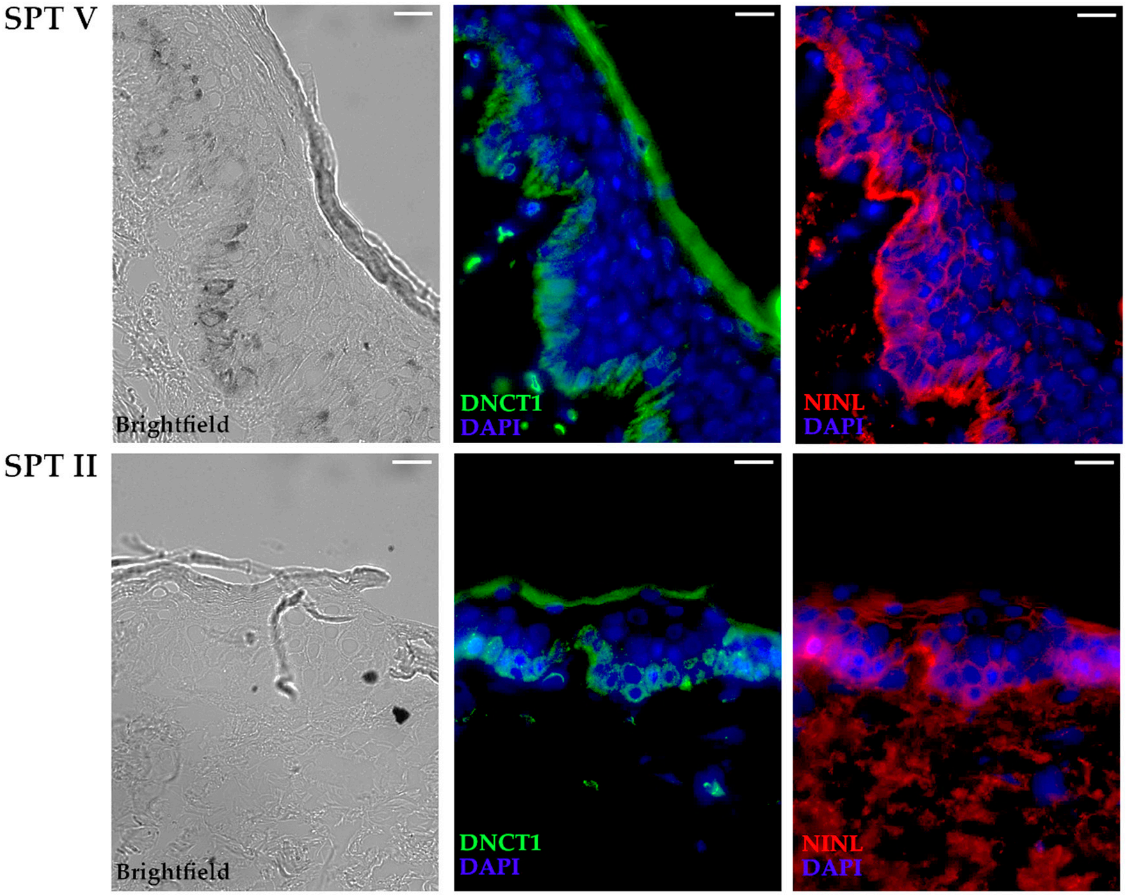

Supplementary Figure S6: Expression of the motor proteins DNCT1 (green) and NINL (red) was broadly similar in both high (SPT V) vs. low (SPT II) pigmented human skin (scale bar = 20 μm).
